# Short-term responses of meat ewes facing an acute nutritional challenge in early-mid lactation

**DOI:** 10.1093/tas/txad141

**Published:** 2023-12-11

**Authors:** Eliel González-García, Marcelo Gindri, Christian Durand, Noëllie Lafon, Sébastien Douls, Gaëtan Bonafe, Valentin Coulon, Dominique Hazard, Laurent Bonnal, Anne Tesnière, Irene Llach, Sara Parisot, Laurence Puillet

**Affiliations:** SELMET, INRAE, CIRAD, L’Institut Agro Montpellier SupAgro, Univ Montpellier, 34060 Montpellier, France; Université Paris-Saclay, INRAE, AgroParisTech, UMR Modélisation Systémique Appliquée aux Ruminants, 91120 Palaiseau, France; INRAE UE321 La Fage, 12250 Saint-Jean-et-Saint-Paul, France; INRAE UE321 La Fage, 12250 Saint-Jean-et-Saint-Paul, France; INRAE UE321 La Fage, 12250 Saint-Jean-et-Saint-Paul, France; INRAE UE321 La Fage, 12250 Saint-Jean-et-Saint-Paul, France; INRAE UE321 La Fage, 12250 Saint-Jean-et-Saint-Paul, France; INRAE UMR1388 GENPHYSE Université de Toulouse, ENVT, 31326 Castanet-Tolosan, France; SELMET, CIRAD, INRAE, L’Institut Agro Montpellier SupAgro, Univ Montpellier, 34398 Montpellier, France; SELMET, INRAE, CIRAD, L’Institut Agro Montpellier SupAgro, Univ Montpellier, 34060 Montpellier, France; SELMET, INRAE, CIRAD, L’Institut Agro Montpellier SupAgro, Univ Montpellier, 34060 Montpellier, France; INRAE UE321 La Fage, 12250 Saint-Jean-et-Saint-Paul, France; Université Paris-Saclay, INRAE, AgroParisTech, UMR Modélisation Systémique Appliquée aux Ruminants, 91120 Palaiseau, France

**Keywords:** adaptive capacity, between-animal differences, lambs’ suckling, resilience’ biomarkers, response-recovery profiles

## Abstract

Simulating a consequence of a climate change event on feed availability, responses of Mediterranean meat ewes facing an acute undernutritional challenge (**CHA**; i.e., fed only low nutritional value cereal straw) were evaluated at a sensitive physiological stage (i.e., early suckling). Forty Romane ewes were chosen at early-mid pregnancy (around 2 mo) according to parity (20 primiparous, **PRIM**; 20 multiparous, **MULT**); feed efficiency genetic line of their sires (residual feed intake [**RFI**]; efficient, RFI**−**, *n* = 10 per parity; inefficient, RFI**+**, *n* = 10 per parity); litter size (i.e., bearing twins, diagnosed by ultrasonography); body weight (**BW**, kg) and body condition score (**BCS**) (initial BW and BCS [mean ± SD]: 51.6 ± 7.41 kg; 2.5 ± 0.20, respectively; representing flock’ averages per parity). Effects on dry matter intake (**DMI**), ewes’ BW and BCS, subcutaneous dorsal fat thickness (**DFT**), energy metabolism (plasma non-esterified fatty acids [**NEFA**], β-hydroxybutyrate (**β-OHB**), glucose, urea, triiodothyronine [**T3**]), and lambs’ growth (BW and average daily gain [**ADG**]; g/d) were examined before, during and after CHA. Individuals’ profiles of the response-recovery to CHA were described using a piecewise mixed-effects model. The fixed effect of parity and genetic line and the random effect of individual (ewe) were considered. A linear mixed-effects model was fitted to explore the effects on lambs’ growth. The 2-d straw-only CHA had significant effects on most of the recorded parameters. Meaningful drops and recoveries were observed on ewes’ DMI, BW, and DFT with effect on postchallenge levels. BW, BCS, DFT, or DMI were also affected by parity (MULT > PRIM) but not by genetic line. Plasma NEFA, β-OHB, glucose, urea, and T3 responded well to CHA with drops in T3, urea, and glucose levels, whereas NEFA and β-OHB significantly increased after CHA. MULT ewes presented sharper β-OHB recovery from CHA than PRIM (*P* ≤ 0.05). With this study, we provide tangible and necessary data for an emerging field of research. Our results give new insights into how such a short and abrupt CHA affects some key zootechnical and physiological parameters, and to what extent the impacts of CHA and the ewes’ response-recovery are influenced. It also revealed potential between-individual differences in the adaptive capacities of ewes, which require further exploration.

## Teaser

Increasing our knowledge about mechanisms displayed by ruminants in response to unpredictable nutritional challenges imposed by climate change is a priority and here we provided quantifiable data for a rich set of zootechnical and physiological parameters, in the responses and recovery of ewes submitted to an acute challenge after lambing. The potential of key short-term indicators (potential biomarkers) such as intake, dorsal fat thickness, and plasma metabolites was proven.

## Introduction

Availability, temporality, and quality of feedstuffs to be offered for animal feeding in livestock farming systems worldwide are being strongly impacted by climate change perturbations, and the future promises even worse scenarios if no drastic solutions are urgently implemented. The nature of animals’ responses to such environmental challenges will be closely related to the local environment (e.g., climatic conditions and seasons), to farm management strategies (e.g., feeding system), but also the individual animal per se, with responses modulated by physiological stages (growth, pregnancy, lactation, etc.) and the specific adaptive capacities determining its potential robustness to cope with such challenges.

To face feed shortages provoked e.g., by long dry seasons or other unpredictable hazards, efficient mechanisms leading to physiological or metabolic adjustments involve orchestrated homeostasis and homeorhesis regulations (Bauman and Currie, 1980; Bauman, 2020). Those related to the administration of energy body reserves (**BR**) in ruminants (i.e., efficient synchronization of BR mobilization-accretion processes) are well known, mainly in harsh environments (Bocquier and González-García, 2010). Short-term responses include adjustments in intake, digestion, and metabolism (Chilliard et al., 1998, 2000; González-García et al., 2020a).

Factors such as parity (and age) and litter size are also well known to be among the main factors driving the adaptive capacity of the female to cope with challenging situations in their productive cycles and careers (Nielsen et al., 2003; Meikle et al., 2004; Friggens et al., 2007; González-García et al., 2014, 2015). The individual adaptive capacity of an animal facing a nutritional challenge is likely to increase with the increase in the frequency for which it is repetitively facing the same challenge. In past works (González-García et al., 2014, 2015, 2020b), we found differences in responses between young and adult reproductive females submitted to undernutrition events, which is likely related to an increase in the maturity of the mechanisms to be displayed as productive life progresses. Similarly, the litter size is a classical factor implicated in the ‘pull effect’ for energy demands, thus affecting energy balance with more or less intensity (i.e., higher energy requirement at higher litter size; González-García et al., 2014, 2015). Results are less clear however with regard to the feed efficiency genetic line, a relatively new trait with less available reports. In a recent work, when evaluating the effects of an acute nutritional challenge in the response and recovery of two feed efficiency genetic lines of Assaf ewes in Spain, Barrio et al. (2023) suggested greater tissue mobilization in more efficient ewes according to the observed changes in blood β-hydroxybutyrate (**β-OHB**). This supports the idea that metabolic adaptation during the underfeeding period was higher (or faster) in high-efficient ewes compared to low-efficient ones. In dairy cows, Potts et al. (2015) also argued that because body energy changes are accounted for in the prediction of residual feed intake (**RFI**), it is expected that low-RFI individuals will not be any more likely to mobilize body tissue to support production than cows with high RFI (efficient). Contrary to those statements, in a previous study carried out by our team (Azizi et al., 2021) we reported no difference in voluntary intake and digestibility in ewes originated from two divergent RFI lines of Romane sheep. As it is an emerging field of research, there is lack of quantifiable, reliable data disentangling the magnitude of the effects of a nutritional challenge in different physiological and productive parameters. Therefore, the objective of this work was to determine the effects of a short-term and acute nutritional challenge (**CHA**) on freshly lambed meat ewes and their offspring. We hypothesized that either the response to or the recovery from that challenge may be immediately reflected in the ewes’ body condition, feed intake rate, the related energy metabolism, and the growth rate of their lambs. The amplitude of such responses could be modulated to some extent by the ewes’ parity, feed efficiency genetic line, litter size at suckling, or the sex of lambs, but also by other potential between-individual differences.

## Materials and Methods

### Experimental site and approval of animal procedures

The study was conducted at the INRAE Experimental Farm *La Fage*, *Causse du Larzac* (43°54’54.52”N; 3°05’38.11”E; https://uef.isc.inrae.fr/), Saint-Jean-et-Saint-Paul (Aveyron, France). Animals handling and care, as well as detailed experimental procedures for each measurement, were approved by the Regional Ethics Committee on Animal Experimentation number 115 (Languedoc-Roussillon, France); file number 2021112407509353, Agreement with reference APAFIS_34105.

### Animals, experimental conditions, feeding and management

The experimental model was the reproductive female of the *Romane* sheep breed from the flock of *La Fage*, which is reared fully outdoor under extensive rangeland conditions (i.e., 280 females in 280 ha; González-García et al., 2014). Forty ewes (initial body weight [**BW**] and body condition score [**BCS**; mean ± SD]: 51.6 ± 7.41 kg; 2.5 ± 0.20, respectively) were chosen at the early-mid pregnancy (around 2 mo) according to parity (20 primiparous, **PRIM**; 20 multiparous, **MULT**), feed efficiency genetic line of their sires (RFI; Tortereau et al., 2020; efficient, RFI−, *n* = 10 per parity; inefficient, RFI+, *n* = 10 per parity), litter size (i.e., diagnosed by ultrasonography on January 25; an effort was made to mainly select ewes with two fetuses), and body condition (i.e., animals representing the average BW and BCS of their respective parity in the flock). Values of these criteria for the 40 retained ewes, at the start of the experiment, are presented in Supplementary Table S1. Ewes originated from two divergent lines of sires genetically selected for RFI (Tortereau et al., 2020). Sires were selected based on their breeding values for RFI (i.e., difference between observed and predicted feed intakes), the average RFI indexes were −92.0 g/d and +75.0 g/d for efficient (RFI−) and inefficient (RFI+) sires, respectively.

After ewes were chosen and identified at the early-mid pregnancy, they stayed in the rangeland until lambing (average lambing date for these ewes was April 10 [±7 d]). Then, in function of their precise lambing date, ewes were progressively moved to the controlled conditions of an indoor facility in which the full experiment was carried out. The indoors housing is a confined pen with straw-bedding (total area of ~80 m²) equipped with individual feeders (*n* = 48; 0.4 m wide × 0.5 m long × 0.4 m depth) and free access to water and mineral salts. A detailed description of this pen (including images) was previously reported (González-García et al., 2018). Each ewe was fitted with electronic identification and recognized by only one individual automatic feeder within the pen during the full experiment, thus allowing individual daily intake to be measured. Due to animal welfare issues (i.e., density or number of individuals per m²) and considering that each ewe was kept with its lambs, from the 48 automatic feeders’ places available in the confined pen only 40 were used.

**Figure 1.**
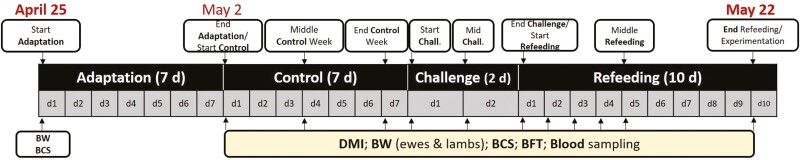
Schematic representation of the experimental design, which tracked a 2-d abrupt nutritional challenge. The challenge was in early lactation, during the suckling period (29 ± 6.8 d after lambing). The experiment lasted around 40 d (from April to May 22), and consisted in i) a preadaptation period allowing ewes to acclimate to the general environment of the housing facility and to be trained to use individual automatic feeders (lasting 7 to 14 d, depending on lambing date which determined the arrival to the experimental pen; not shown in the figure); ii) an adaptation week (7 d; April 25 to May 1); iii) a control week (7 d; May 2 to 9); the only straw nutritional challenge (2 d; May 10 and 11); and iv) a refeeding or recovery period back to the hay (10 d; May 12 to 22).

Except during the 2-d-only-straw challenge (Figure 1), since the start of the adaptation period, and during the control and refeeding weeks, the diet was based on first-cut, high-quality chopped hay offered ad libitum and composed of ~50% alfalfa (*Medicago sativa*), 35% cocksfoot (*Dactylis glomerata*), and 15% ryegrass (*Lolium perenne*; Table 1). During the first 2 wk of acclimation, the hay was distributed to the whole group in a belt feeder. Once ewes were adapted to the new feeding regime, the chopped hay was then offered within individual feed bins placed on top of the belt feeder used previously. The hay was offered twice daily at 0900 and 1700 hours (60% and 40% of the allowance, respectively). During the final week of the adaptation phase (days 15 to 20, once ewes were well-adapted), measurement of daily individual feed intake started. The cereal straw distributed during the challenge was also chopped at a similar length to the hay. The automatic feeding system allowed adjustment to a precise quantity of feed to offer for each ewe on each day in accordance with its intake level. The allowance was continually adjusted to 115% of the previous day’s voluntary intake. Ewes also had continuous access to fresh water and mineral salts.

**Table 1.** Nutritive value of the first-cut, high-quality hay comprising 50% alfalfa (*Medicago sativa*), 35% cocksfoot (*Dactylis glomerata*) and 15% ryegrass (*Lolium perenne*) fed to ewes ad libitum since the start of the adaptation period, and during the control and refeeding weeks of the experiment. Data for the low quality cereal straw distributed during the 2-d nutritional challenge are also shown

Nutrient	Hay	Straw
DM, g/kg as fed	945	945
Organic constituents, g/kg DM		
OM	910	968
CP	156	24
CF^1^	369	447
NDF	536	821
ADF	363	464
ADL	83	61
IVDMD^2^	570	238
IVOMD^3^	539	237
Estimated net energy for lactation^4^, Mcal/kg DM	1.07	0.59
Protein value, g/kg DM		
DPIN^5^	112	18
DPIE^6^	90	36
Fill value, FUV^7^	1.14	2.51

^1^Crude fiber content.

^2^ *In vitro* DM disappearance.

^3^ *In vitro* OM disappearance.

^4^Net energy forage unit for lactation (FUL). In INRAE system: *Unité Fourragère du Lait* (UFL); 1 UFL = 1.7 Mcal of FUL.

^5^Digestible proteins in the intestine when nitrogen (N) is limiting, calculated as: PDIA (dietary protein undegraded in the rumen which is digestible in the small intestine) + PDIMN (microbial protein that can be synthesized from the rumen degraded dietary N when energy is not limiting).

^6^Digestible proteins in the intestine when energy is limiting, calculated as: PDIA + PDIME (microbial protein that can be synthesized from the energy available in the rumen when degraded N is not limiting).

^7^Forage fill unit value for sheep (Jarrige, 1988).

### Experimental design

A schematic representation of the experimental design is provided in Figure 1, which followed the 2-d abrupt nutritional challenge (underfeeding model) proposed by Friggens et al. (2016). The challenge was in early-mid lactation, during the suckling period (29 ± 6.8 d after lambing). The experiment lasted around 40 d (from early April to May 22), and consisted in: i) a preadaptation period allowing ewes to acclimate to the general environment of the housing facility and to be trained to use the electronic individual automatic feeders (lasting 7 to 14 d, depending on lambing date which determined the arrival to the experimental pen); ii) an adaptation week (7 d; April 25 to May 1); iii) a control week (7 d; May 2 to 9); iv) the straw-only CHA (2 d; May 10 and 11); and, v) a refeeding or recovery period back to the normal diet (10 d; May 12 to 22).

The most important part of the experimental design is undoubtedly the straw-only CHA to which the ewes were subjected (Figure 1). It consisted of feeding all the ewes, for two consecutive days, with a very low-quality cereal straw (chopped in the same way as hay). The feeding system management routine during that CHA was identical to the rest of the days.

### Measurements, sampling procedures, and analyses

The schedule followed for all measured parameters is also shown in Figure 1. Ewes were monitored for their daily individual feed intake, body condition (BW and BCS), dorsal fat thickness (**DFT**), and plasma profiles of non-esterified fat acids (**NEFA**), glucose, β-OHB, urea, and triiodothyronine metabolic hormone (**T3**). Individual feed intake was measured daily since the adaptation week. BW, BCS, DFT, and plasma profiles of ewes, as well as BW of lambs, were measured 11 times around CHA (i.e., before, during, and after the 2-d only straw distribution).

During the first weeks (preadaptation period), feed intake was monitored on a fresh matter basis. From the start of the adaptation week until the end of the experiment, hay (or straw) dry matter (**DM**) was determined (on oven-dried samples at 65 °C during 48 h) and feed refusals were registered every morning, so daily individual DM intake (**DMI**) was recorded. The DMI was expressed as total (g DM/ewe/d) or in function of the ewe’s BW (g DM/kg BW) or metabolic BW (g DM/kg BW^0.75^).

BW and BCS were recorded at the start and at the end of the experiment, at the start of the adaptation period; at the start, middle, and end of the control week; then, daily during eight consecutive days from the day before, during, and after the 2-d only straw challenge (Figure 1). The static ewes’ BW measurements were performed using conventional scales with the help of a Combi clamp (Ritchie Agricultural, Angus, Scotland), at which time the BCS was assessed by a trained operator according to an adaptation of the original grid described by Russel et al. (1969) which was further divided into a 1/10 scale, i.e., from 1 to 5 with 0.1 increments. The ewes’ DFT (cm) was measured by ultrasonography of dorsal fat layer using an Easi-Scan Linear portable scanner (BCF Ultrasound Australasia, Victoria, Australia). The dorsal region of each ewe was previously shaved to facilitate scanning and ensure image quality.

In lambs, BW were also measured during the morning of the 11 points of measurements for the ewes (i.e., before, during, and after the 2-d only straw CHA), to assess potential effects of the ewe feeding status, through indirect impact on milk yield and lamb growth (assessed by calculation of ADG, g/d).

Plasma was sampled during the same dates (*n* = 11 samples/ewe) to determine metabolic profiles of physiological traits associated with BR mobilization and accretion at the start, mid-point, and end of the Control week; and before, during, and after CHA. Blood was sampled by jugular venipuncture before the first meal at ~0800 hours on each sampling day. Two 9 mL blood samples were drawn from each ewe by a trained operator; one tube with 18 IU of lithium heparin per 1 mL blood, and another with 1.2 to 2 mg of potassium EDTA per 1 mL blood (Vacuette Specimen Collection System, Greiner Bio-One GmbH, Austria). Samples were immediately placed on ice before centrifugation at 3,600 × *g* for 20 min at 4 °C. The plasma was collected and stored at −20 °C in individually identified aliquots of 3 µL for metabolite and hormone analyses. Plasma concentrations of NEFA, glucose, β-OHB, urea, and T3 were determined according to the protocols described by González-García et al. (2014, 2015).

Plasma NEFA concentration was measured in duplicate using the commercially available Wako NEFA-HR(2) R1 and R2 kit (Fujifilm Wako Chemicals GmbH, Neuss, Germany) adapted for 96-well microplates. Intra- and inter-assay variation averaged 3.29% and 2.51%, respectively. Plasma glucose concentration was measured in triplicate using a commercially available glucose GOD-PAP kit (reference LP87809; manufactured and distributed by Biolabo SAS, Maizy, France) adapted for 96-well microplates. Intra- and inter-assay variation averaged 2.63% and 2.30%, respectively. Plasma concentration of β-OHB was measured in triplicate using a commercially available 3-Hydroxybutyrate Dehydrogenase (3-HBDH) kit (Roche Diagnostics Deutschland GmbH, Mannheim, Germany). Intra- and inter-assay variation averaged 2.51% and 2.49%, respectively. Plasma concentration of urea was measured in triplicate using a commercially available Urea UV kit (manufactured and distributed by Biolabo SAS). Intra- and inter-assay variation averaged 2.11% and 1.24%, respectively. Plasma concentration of T3 was measured in duplicate using a commercially available T3 (Triiodothyronine) ELISA kit (manufactured by NeoBiotech, Nanterre, France). Intra- and inter-assay variation averaged 5.04% and 4.94%, respectively.

The daily feed that was distributed and the refusals were daily weighed and sampled individually to determine feed intake. The DMI was calculated as the difference between the quantity of DM offered and refused. The organic matter (OM) content (%) was calculated as the difference between 100 and the percentage of ash in each sample. Table 1 shows the chemical composition and nutritive value of the hay and the cereal straw offered during the experiment. Samples were taken twice daily at each distribution in the morning and evening, respectively. Feed refusals were weighed every morning before the first meal was distributed. Samples (10%) were collected and dried at 60 °C for 48 h to determine DM content and, at the end of the trial, were milled through a 1-mm screen in a hammer mill and stored for further analysis in the laboratory.

As described by Azizi et al. (2021), the chemical composition of the dried and ground feed and refusals samples (hay and straw) were determined by monochromatic NIRS (NIRS 6500, Foss NIRSystems, Silver Spring, MD, USA). The chemical composition was predicted based on its NIRS spectrum using reference data (SELMET-CIRAD, Montpellier, France) derived from a large sample population collected over multiple years in two databases. The parameters considered were CP (Kjeldahl method), fiber fractions (NDF, ADF, ADL; method number 973.18; Van Soest et al., 1991), and in vitro DM and OM digestibility (Aufrère et al., 2007). The net energy forage unit for lactation (**FUL**), digestible proteins in the intestine when nitrogen (**DPIN**) or energy (**DPIE**) are limiting, and the forage fill value for sheep (**FFV**) were calculated using INRAE’s PrevAlim software (Baumont et al., 1999).

### Data processing and statistical analyses

#### Investigating the effect of ewes’ genetic line and parity

The data daily recorded of ewes’ BW (kg), DMI (kg/d; g/kg BW; and g/kg BW^0.75^), plasma metabolites, and T3 were analyzed using a piecewise approach. The responses/recoveries profiles of these traits to the CHA were analyzed using a piecewise mixed-effects model (adapted from Friggens et al., 2016), which was fitted with four parameters that describe the phases of the experimental challenge: V1, the model intercept that describes the prechallenge period; V2, linear rate of response to 2-d CHA; and, V3 and V4, that represent the linear rate of recovery from challenge and quadratic rate of deceleration in recovery from challenge, respectively, which lasted until day 6 from refeeding-confirmed by visual inspection of data. In summary, as described by Friggens et al. (2016), the rate of response to CHA means the linear slope of the ewes’ response during the 2-d CHA (V_2_ coefficient); the rate of recovery from CHA is the linear component of the ewes’ recovery to the 2-d CHA (V_3_ coefficient), whereas the rate of deceleration on recovery is the quadratic component (deceleration) of the ewes’ recovery to the 2-d CHA (V_4_ coefficient).

The fixed effects affecting the features of experimental ewes (i.e., genetic line and parity) and the random effect of individuals (i.e., ewe) were considered in all four parameters of the piecewise mixed-effects model. The random effects were assumed to be ~iidN(0,σ_B_^2^). The residual error was assumed to be ~N (0, R), with R as the heterogeneous autoregressive of order 1 error covariance structure, used to correct for lack of independence in the residual and to correct heterogeneity of variances along predictions. The *lme* function of the *nlme* package (Pinheiro and Bates, 2000) of software R (R Core Team, 2022) was used to fit the piecewise models. Contrasts on the models’ parameters, using general hypothesis testing, function *glht* of package *multcomp* (Hothorn et al., 2008) of R (R Core Team, 2022), were used to evaluate interactions when significant, and the stabilization period (i.e., postchallenge, *V*5) recovery and to test differences between prechallenge and stabilization periods. The contrasts to calculate the postchallenge period were set according to the following equation:


$$\begin{array}{l} V5\;=V1\;+\;V2\;\times \;2\;+\;V3\;\times \;4\;+\;V4\;\times \;4^{2} \end{array}$$

All the graphics were performed using *ggplot2* package of R (R Core Team, 2022). Statistical significance was set at *P* ≤ 0.05.

To explore the effect of the ewes’ genetic line and parity on lambs’ BW (kg) and lambs’ ADG during the experiment, we fitted a linear mixed-effects model. For this, we considered the fixed effects of days of experiment, genetic line, parity, lambs’ sex, litter size at suckling, the interaction genetic line × parity, lambs’ sex × parity and litter size at suckling × parity and the random effects of lamb nested in ewe [assumed to be ~ iidN(0, σ_B_^2^)] and residual error, that was assumed to be ~ N (0, R), with R as the heterogeneous autoregressive of order 1 error covariance structure, used to correct for lack of independence in the residual and to correct heterogeneity of variances along predictions.

## Results

### Short-term effects of 2-d nutritional challenge on ewes’ response/recovery

**Figure 2.**
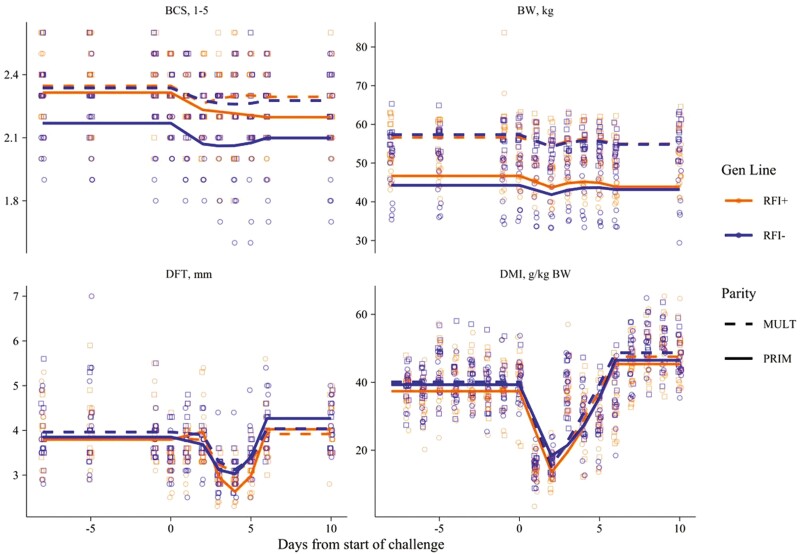
Overall trends in BW, BCS, DFT, and DMI of Romane ewes (primiparous, PRIM, or multiparous, MULT; efficient, RFI−, or inefficient, RFI+), when facing a 2-d nutritional challenge during suckling (i.e., 29 ± 6.8 d after lambing).

**Figure 3.**
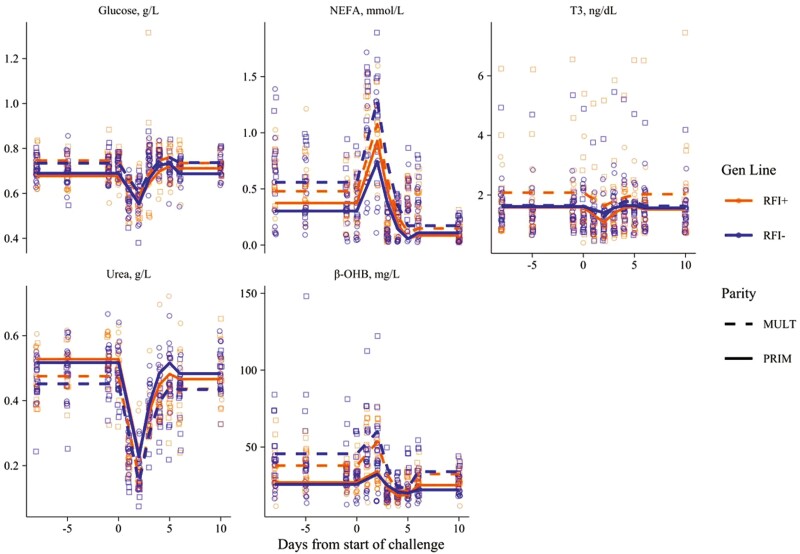
Overall trends in plasma concentrations of β-OHB, Glucose, NEFA, T3 and Urea of Romane ewes (primiparous, PRIM, or multiparous, MULT; efficient, RFI−, or inefficient, RFI+), when facing a 2-d nutritional challenge during suckling (i.e., 29 ± 6.8 d after lambing).

The average time trends for the ewes’ parameters DMI, body condition (BW and BCS), and subcutaneous DFT, as well as for plasma metabolites and T3 are presented in Tables 2 and 3; and Figures 2 and 3, respectively. As expected, the 2-d straw-only feeding caused a significant effect (*P* < 0.001) in most of the recorded parameters. Meaningful drops and recoveries were observed on ewes’ DMI, BW, and DFT with effect (*P* < 0.001) on the postchallenge levels (Figure 2 and Supplementary Figures S1 to S6). The BW, BCS, DFT, or DMI was not affected (*P* > 0.05) by genetic line or the interaction of this factor with the ewe’ parity (Table 3). These traits were only influenced to some extent by parity. As expected, MULT ewes presented higher values compared to PRIM ewes for BW (*P* ≤ 0.001), total DMI (kg/d; *P* ≤ 0.001), DMI per metabolic BW (g DM/kg BW^0.75^; *P* ≤ 0.048) during the prechallenge and DMI rate of response to challenge (kg/d; *P* ≤ 0.020; Table 3). The ewes’ parity significantly affected the DFT rate of recovery from CHA and the DFT rate of deceleration on recovery (Table 3).

**Table 2.** Average time trends for DMI (kg or g), BW (kg), BCS (1 to 5 point scale), and DFT (cm) of Romane ewes from two divergent feed efficiency genetic lines and parity (multiparous [MULT], primiparous [PRIM]), before, during, and after a 2-d acute nutritional challenge

	PRIM		MULT		SEM	Effect, *P* value		
	RFI−	RFI+	RFI−	RFI+		Parity	Line	Parity × Line
DMI, kg/d								
Prechallenge level	1.78	1.74	2.32	2.30	0.094	<0.001	0.911	0.8852
Rate of response to challenge	−0.585	−0.479	−0.726	−0.728	0.043	0.020	0.969	0.2093
Rate of recovery from challenge	0.274	0.182	0.334	0.445	0.120	0.724	0.510	0.3965
Rate of deceleration on recovery	0.019	0.033	0.027	0.006	0.029	0.851	0.608	0.5458
Postchallenge level^1^	2.02	2.04	2.63	2.72	—	—	—	—
DMI, g/kg of BW								
Prechallenge level	37.40	39.31	40.12	40.08	1.575	0.229	0.985	0.5383
Rate of response to challenge	−11.91	−10.48	−12.27	−12.43	0.780	0.746	0.879	0.3057
Rate of recovery from challenge	5.25	1.81	5.53	7.23	2.368	0.936	0.611	0.2777
Rate of deceleration on recovery	0.67	1.31	0.62	0.29	0.585	0.944	0.690	0.4112
Postchallenge level	45.38	46.51	47.54	48.70	—	—	—	—
DMI, g/ kg metabolic BW (BW^0.75^)								
Prechallenge level	98.32	100.63	110.54	110.46	4.223	0.048	0.990	0.7791
Rate of response to challenge	−31.58	−26.62	−34.01	−34.47	2.040	0.399	0.873	0.185
Rate of recovery from challenge	14.3	7.24	15.6	20.4	6.303	0.886	0.589	0.3472
Rate of deceleration on recovery	1.55	2.65	1.55	0.63	1.551	0.999	0.675	0.5148
Postchallenge level	117.1	118.77	129.6	133.2	—	—	—	—
BW, kg								
Prechallenge level	46.69	44.27	56.63	57.35	1.545	<0.001	0.744	0.3164
Rate of response to challenge	−1.50	−1.22	−1.10	−1.56	0.201	0.168	0.106	0.0663
Rate of recovery from challenge	1.44	1.42	1.63	1.40	0.369	0.716	0.659	0.7705
Rate of deceleration on recovery	−0.35	−0.27	−0.37	−0.31	0.084	0.836	0.611	0.9306
Postchallenge level	43.88	43.17	54.97	54.82	—	—	—	—
BCS, 1 to 5								
Prechallenge level	2.31	2.17	2.35	2.34	0.0460	0.6273	0.8812	0.1485
Rate of response to challenge	−0.0413	−0.0495	−0.0416	−0.0300	0.0152	0.9865	0.5898	0.5159
Rate of recovery from challenge	−0.0082	−0.0150	0.0272	−0.0175	0.0332	0.452	0.3415	0.5682
Rate of deceleration on recovery	−0.00012	0.00555	−0.00484	0.00437	0.0079	0.6724	0.4091	0.8225
Postchallenge level	2.20	2.10	2.29	2.28	—	—	—	—
DFT, mm								
Prechallenge level	3.80	3.85	3.84	3.96	0.160	0.8425	0.5915	0.8369
Rate of response to challenge	0.0857	−0.0831	−0.0284	−0.0300	0.093	0.3839	0.9903	0.3672
Rate of recovery from challenge	−1.353	−0.804	−0.747	−0.816	0.188	0.0235	0.797	0.1017
Rate of deceleration on recovery	0.342	0.237	0.195	0.212	0.045	0.0205	0.7861	0.1723
Postchallenge level	4.02	4.26	3.92	4.04	—	—	—	—

^1^For all traits but BCS of MULT × RFI + and DFT of MULT and PRIM RFI + the postchallenge level was different from the prechallenge level (*P* ≤ 0.037). The postchallenge level of DMI (kg/d) and BW were different between MULT and PRIM regardless of genetic line (*P* ≤ 0.001). The postchallenge level of DMI (g/kg BW) and DFT were not affected by parity or genetic line (*P* ≥ 0.141). The MULT × RFI− presented DMI (g/kg BW^0.75^) at postchallenge different to PRIM (*P* ≤ 0.047). The PRIM × RFI− presented BCS postchallenge different from MULT (*P* ≤ 0.047).

**Table 3.** Average time trends for plasma non-esterified fat acids (NEFA), β-hydroxybutyrate (β-OHB), glucose, triiodothyronine (T3), and urea of Romane ewes from two divergent feed efficiency genetic lines and parity (multiparous [MULT], primiparous [PRIM]), before, during, and after a 2-d acute nutritional challenge

Trait	PRIM		MULT		SEM	Effect, *P* value		
	RFI−	RFI+	RFI−	RFI+		Parity	Line	Parity × Line
NEFA, mmol/L								
Prechallenge level	0.303	0.374	0.558	0.479	0.0620	0.040	0.372	0.233
Rate of response to challenge	0.219	0.277	0.363	0.299	0.0442	0.719	0.307	0.171
Rate of recovery from challenge	−0.440	−0.530	−0.649	−0.587	0.0839	0.632	0.602	0.366
Rate of deceleration on recovery	0.0705	0.0799	0.0928	0.0887	0.0154	0.686	0.851	0.661
Postchallenge level^1^	0.109	0.085	0.172	0.148	—	—	—	—
β-OHB, mg/L								
Prechallenge level	25.7	27.0	45.6	37.9	2.87	0.011	0.066	0.126
Rate of response to challenge	3.31	3.59	7.21	7.73	1.92	0.129	0.848	0.951
Rate of recovery from challenge	−8.9	−13.6	−29.6	−26.8	3.82	0.015	0.612	0.334
Rate of deceleration on recovery	1.60	2.84	5.76	5.39	0.72	0.012	0.718	0.264
Postchallenge level	22.1	25.2	33.9	32.4	—	—	—	—
Glucose, g/L								
Prechallenge level	0.690	0.677	0.734	0.746	0.0124	<0.001	0.505	0.340
Rate of response to challenge	−0.0685	−0.0569	−0.0664	−0.0821	0.0106	0.095	0.297	0.200
Rate of recovery from challenge	0.140	0.098	0.109	0.125	0.0202	0.340	0.569	0.150
Rate of deceleration on recovery	−0.0266	−0.0153	−0.0188	−0.0218	0.00436	0.293	0.633	0.103
Postchallenge level	0.687	0.711	0.737	0.735	—	—	—	—
T3, ng/dL								
Prechallenge level	1.61	1.58	1.65	2.08	0.354	0.327	0.396	0.519
Rate of response to challenge	−0.106	−0.250	−0.194	−0.226	0.0592	0.769	0.701	0.344
Rate of recovery from challenge	0.205^ab^	0.378^ab^	0.423^a^	0.192^b^	0.089	0.139	0.066	0.023
Rate of deceleration on recovery	−0.0410^ab^	−0.0674^ab^	−0.0828^a^	−0.0230^b^	0.0215	0.145	0.050	0.046
Postchallenge level	1.57	1.52	1.63	2.03	—	—	—	—
Urea, g/L								
Prechallenge level	0.517	0.528	0.452	0.475	0.0195	0.065	0.401	0.745
Rate of response to challenge	−0.145	−0.145	−0.153	−0.157	0.0100	0.363	0.738	0.802
Rate of recovery from challenge	0.193	0.154	0.177	0.168	0.0206	0.623	0.778	0.452
Rate of deceleration on recovery	−0.0323	−0.0243	−0.0262	−0.0249	0.0048	0.932	0.848	0.487
Postchallenge level	0.483	0.466	0.434	0.436	—	—	—	—

^1^The postchallenge level was different than prechallenge level for NEFA regardless genetic line and parity, β-OHB of MULT, glucose of PRIM RFI+, and urea of PRIM and MULT × RFI + (*P* ≤ 0.021). The postchallenge level was similar to prechallenge level for T3 regardless genetic line and parity, β-OHB of PRIM, glucose of MULT and PRIM × RFI−, and urea MULT × RFI− (*P* ≥ 0.217).

For all performance traits but BCS of MULT × RFI + ewes and DFT of MULT and PRIM × RFI + ewes, the postchallenge level was different from prechallenge (*P* ≤ 0.037). The postchallenge levels of DMI (kg/d) and BW were different between PRIM and MULT regardless of genetic line (*P* ≤ 0.001). The postchallenge levels of DMI (g/kg BW) and DFT were not affected by parity or genetic line (*P* ≥ 0.141). The MULT × RFI− ewes presented DMI (g/kg BW^0.75^) at postchallenge different to PRIM (*P* ≤ 0.047). The PRIM × RFI− ewes presented BCS postchallenge different from MULT (*P* ≤ 0.047).

The recorded plasma metabolites (NEFA, β-OHB, glucose, urea) and T3 levels responded to the 2-d CHA (Table 3). Meaningful drops (or rises) and recoveries were observed with effects on the postchallenge levels. Plasma T3, urea, and glucose levels dropped whereas NEFA and β-OHB increased during the 2-d CHA (Figure 3). Plasma NEFA and urea prechallenge levels and response/recovery to CHA were not affected by parity and/or genetic line (*P* ≥ 0.065). Plasma β-OHB and glucose during the prechallenge showed higher levels for MULT compared to PRIM (*P* ≤ 0.011). Plasma β-OHB recovery from CHA was sharper for MULT than PRIM (*P* ≤ 0.015) whereas plasma T3 recovery from CHA was also sharper for MULT × RFI− than others. The postchallenge level was different from prechallenge level for NEFA (regardless of genetic line and parity), β-OHB of MULT, glucose of PRIM × RFI+, and urea of PRIM and MULT × RFI + (*P* ≤ 0.021; Table 3, Figure 3).

By visual inspection, there is evidence of between-animal variability in the response-recovery profiles to the induced short-term CHA (see Figure 4 for NEFA profiles and Supplementary Figures S1 to S11) which deserves further research. Deeper analyses and interpretation in that regard are carried out and presented by these authors in a companion article to be published elsewhere (Gindri et al., 2023).

**Figure 4.**
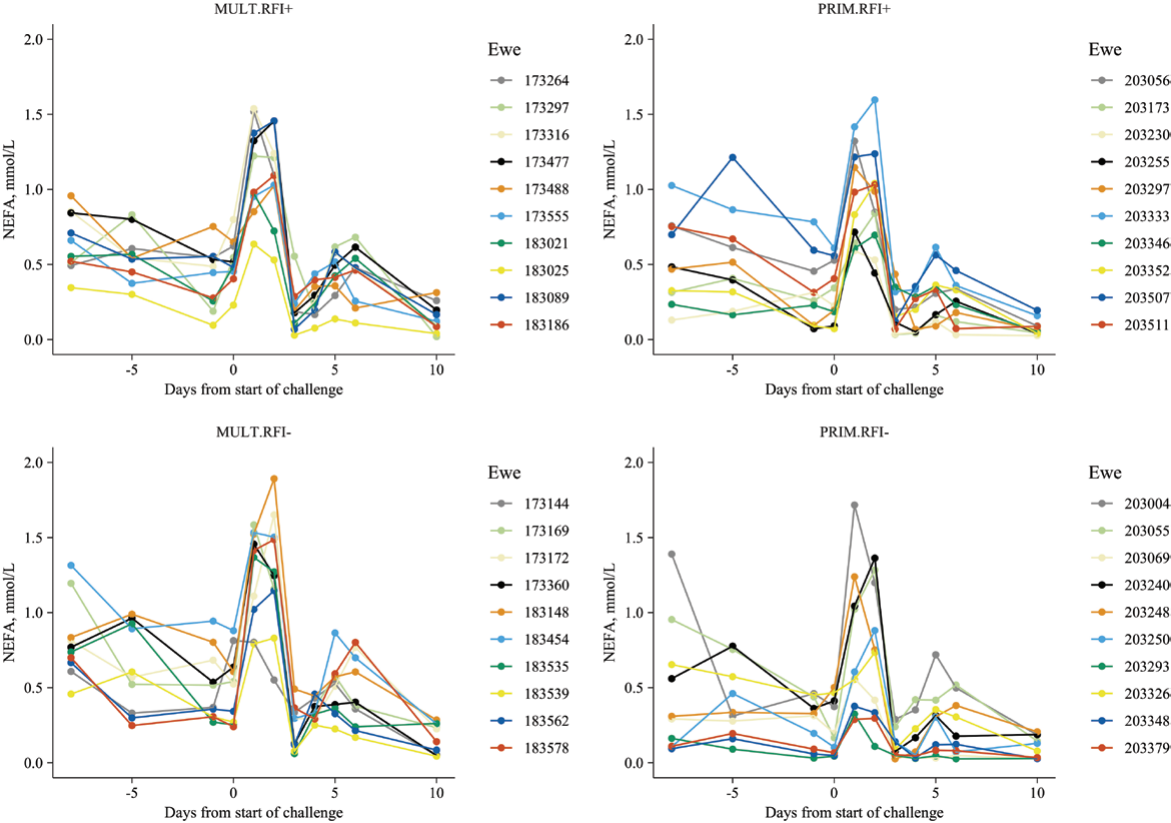
Time trends of the individual response-recovery profiles for plasma NEFA of Romane ewes (primiparous, PRIM, or multiparous, MULT; efficient, RFI−, or inefficient, RFI+), when facing a 2-d nutritional challenge during suckling (i.e., 29 ± 6.8 d after lambing).

### Short-term effects of 2-d nutritional challenge on lambs’ response/recovery

**Figure 5.**
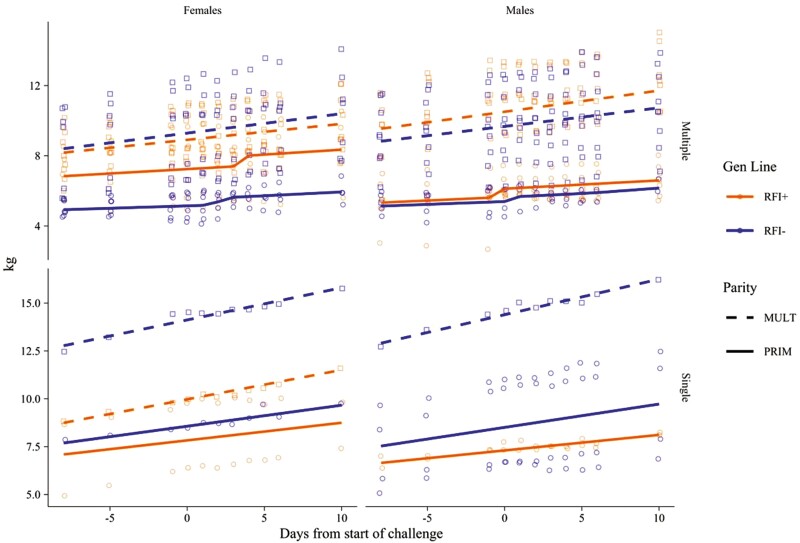
Liveweight progress of suckling lambs (females or males; singletons, SING, or twins, TWIN) from Romane ewes (primiparous, PRIM, or multiparous, MULT; efficient, RFI−, or inefficient, RFI+) facing a 2-d nutritional challenge at around 1-mo after lambing.

Results for lambs’ BW and ADG around the 2-d CHA are presented in Table 4. Significant effects (*P* ≤ 0.001) of ewes’ parity and litter size were observed for both traits. Lambs from MULT ewes presented higher BW and ADG than those from PRIM (*P* ≤ 0.001). No effects were observed on the lambs BW and ADG (*P* ≥ 0.109) due to the interactions parity × genetic line, parity × litter size, and parity × sex, or the isolate effect of genetic line and lamb’ sex. Lambs from MULT ewes presented higher BW at the beginning of the CHA and grew faster (higher ADG) during the experiment than lambs from PRIM ewes (*P* < 0.001). Singletons lambs presented higher BW and ADG during the experiment than lambs from multiple litter size during suckling (*P* < 0.001; Table 4; Figure 5).

**Table 4.** Average BW and ADG of suckling lambs from Romane ewes belonging to two divergent feed efficiency genetic lines and parity (primiparous [PRIM], multiparous [MULT]), after following a 2-d acute nutritional challenge

Trait	Parity		Line		Litter size		Sex		SEM	*P* value			
	PRIM	MULT	RFI−	RFI+	Single	Multiple	Male	Female		Parity	Line	Litter size	Sex
BW lamb													
Intercept, kg	6.9	10.9	8.8	9.0	10.2	7.7	9.1	8.7	0.74	<0.001	0.768	<0.001	0.417
ADG, g/d	70	139	105	104	137	72	109	100	0.02	<0.001	0.885	<0.001	0.383

The interactions parity × line, parity × litter size, and parity × sex were not significant (*P* ≥ 0.109).

## Discussion

### Effectiveness of the model and biomarkers to reflect short-term undernutrition effects

The main purpose of the present study was to evaluate the short-term effects of a CHA with cereal straw only (replacing a good quality hay) in freshly lambed Romane meat ewes suckling their lambs. The piecewise mixed model used here performed well to characterize the short-term temporal patterns and the differences in profiles (Supplementary Figures S1 to S11) of the monitored traits at the prechallenge, during the challenge, and postchallenge stages, according to the 4 parameters to estimate relative to the number of measures in a profile (*n* = 18). Overall, our findings and trends are in agreement with those reported in other works using different animal species or breeds but testing a similar short-term only-straw restriction CHA (i.e., Friggens et al. (2016) and Ben Abdelkrim et al. (2021) in dairy goats; Barrio et al. (2023) in dairy ewes).

By visual inspection, and statistical evidence (*P* < 0.001; see Figures 2 and 3, and Supplementary Figures S1 to S11), we may conclude that DMI, DFT, and plasma NEFA, glucose, and urea were the best indicators to clearly represent the overall expected impacts (drops and/or rebounds) on the experimental ewes during and after the induced short-term CHA. Temporal trends in BW, BCS, and plasma T3 were less sharp. This finding confirms the potential of the aforementioned parameters to be used as reliable biomarkers of the short-term responses of ruminants when facing acute undernutrition events (Chilliard et al., 1998; Ingvarsten and Andersen, 2000; González-García et al., 2020a). Direct effects were not reflected however in the shape of lambs’ BW and ADG curves (Figure 4), demonstrating the capacity of these Romane ewes for responding to the acute CHA without affecting offspring performance in the short-term. We speculate different trends could be expected under longer nutritional restrictions (i.e., with possible sharper drops in the BW and ADG of lambs).

The adaptive capacity of animals to overcome environmental challenges relies on their physiological reorganization capacity to cope with disturbance. The use of body lipids reserves is one of the strategies of domestic ruminants to face with feed shortage situations (Chilliard et al., 2000). Our results demonstrated an increase in plasma NEFA concentration during CHA and a drop in glucose. This indicates the increase of lipolysis during the 2 d feed shortage, along with the reduction of fatty acid re-esterification and reduction in glycerol 3-phosphate synthesis from glucose (Dunshea et al., 1990; Forest et al., 2003). During feed shortage, glucose becomes a scarce metabolite for survival and maintaining lactose synthesis and milk production (Bauman, 2020). Along with this, β-OHB also increased during the nutritional CHA (even if with a less sharp trend) demonstrating incomplete β-oxidation of mobilized NEFA by the liver, which is used as an oxidizable substrate in specific tissues to spare blood glucose (Heitmann et al., 1987). Similar results were also demonstrated by studies with dairy cows and dairy goats during early, mid, and late lactation (Bjerre-Harpøth et al., 2012; Friggens et al., 2016; Billa et al., 2020; Ithurbide et al., 2023).

Temporal patterns in the postchallenge period (ability to revert to the initial status) were well described for DMI, DFT, and plasma NEFA, β-OHB, urea, and T3 which showed return to initial plateau around 4 to 5 d after finishing the CHA (Figures 2 and 3; Supplementary Figures S1 to S11). The potential of NEFA, β-OHB, and T3 as good biomarkers of BR mobilization-accretion processes in sheep is well known and has been reported previously with meat and dairy ewes at La Fage with different rearing systems (González-García et al., 2014, 2015) and beyond (e.g., with Mérinos d’Arles meat ewes; González-García et al., 2020a). Our results are in agreement with the results from Barrio et al. (2023) who found DMI and milk yield values at a steady level of 5 d after the end of the CHA in dairy ewes. They are also concordant with Zou et al. (2019) who reported better efficiency in the energy and protein utilization during refeeding after a feed deprivation period in yaks.

### Effects of feed efficiency genetic line and other key factors (parity, litter size, and lamb’ sex) on the response to and recovery from the challenge

Feed efficiency genetic line (based on RFI) was one of the main fixed factors considered in this study. Our Romane meat ewes were offspring of sires divergently selected for low versus high RFI (Tortereau et al., 2020). The hypothesis was that potential collateral impacts of such genetic selection for feed efficiency (RFI) could have affected the ewes’ resilience, which might be reflected in the response and/or recovery to the CHA event. Our results reject such hypothesis as no difference was found between the two genetic lines for any of the monitored indicators (Tables 2 and 3; Figures 2 and 3). These findings are in agreement with a previous study carried out by our team reporting no difference in voluntary intake and digestibility in ewes originated from the two divergent RFI lines of Romane sheep (Azizi et al., 2021). Our findings disagree with those from a recent report by Barrio et al. (2023) when evaluating effects of a similar short-duration CHA in the response and recovery of two feed efficiency genetic lines of Assaf ewes in Spain. These authors suggested greater tissue mobilization in more efficient ewes according to their observed changes in blood β-OHB, which supports the idea that metabolic adaptation during the underfeeding period was higher (or faster) in high-efficient ewes compared to low-efficient.

In pregnant dairy cows, Fitzsimons et al. (2014) reported a reduction in backfat thickness in low-RFI (efficient) cows, suggesting higher BR mobilization to meet their nutritional requirements compared to high-RFI cows. Potts et al. (2015) also argued that because body energy changes are accounted for in the prediction of RFI, it is expected that cows with low RFI will not be any more likely to mobilize body tissue to support production than cows with high RFI (efficient). The independence of RFI from BW loss is important because excessive tissue mobilization can lead to negative energy balance, which is related to metabolic diseases and poor fertility.

Here again, we might speculate that different results could be expected under longer undernutrition situations i.e., where differences between efficient and inefficient ewes could be more likely to be revealed. The rationale behind this suggestion may be related to the level of synchronization and/or interdependency of biological mechanisms responsible for these two different but somehow inter-related traits (feed efficiency and individual robustness or resilience). A higher feed efficiency may mainly rely, first, on mechanisms related to feed selection and acquisition (determining quantity and quality of the ingested diet), which is impacted by feeding behavior determining ingestion pace and other features (e.g., biting rate, mastication, salivation, regurgitation, and rumination). Then, secondly, a set of factors determining more or less efficient digestion processes (and digestibility of nutrients) at the rumen (e.g., microbial population and ruminal efficiency) and post-ruminal levels, until nutrient absorption. On the other hand, mechanisms related to higher individual resilience or robustness are probably more complex and may depend on interplays occurring at metabolic levels and the capacity of an individual to make priorities. These would underpin the ability to achieve the most optimal nutrient allocation and the most relevant trade-offs between biological functions at a given moment. Nevertheless, it is important to remind that genetic lines for feed efficiency in this Romane sheep are selected during the growth period (lambs) and based on the concentrate fraction of the diet. Up to date, we do not have any evidence of keeping the divergence of the genetic lines when adult animals are reared under different conditions i.e., using roughages (as was discussed by Azizi et al., 2021).

Further research is needed to continue elucidating such complex mechanisms and confirm (or refute) our global hypothesis that the most efficient animal (RFI−) it is not necessarily the most resilient one when facing undernutrition events. The nature of response (and convergences) of these traits may probably depend on the magnitude (intensity) and length of the nutritional CHA. Studies are currently in progress in the Romane genetic lines selected for feed efficiency to confirm that the divergent lines still differed for feed efficiency in productive ewes fed with forages while the selection is applied in growing lambs fed concentrates. Last, we cannot exclude that the absence of genetic effect in the present study may be due to insufficient divergence in feed efficiency in the two group of ewes (i.e., not directly selected for feed efficiency but originated from divergently selected sires) even if sires transmitted to their offspring half of their genetic values for low or high RFI and that divergence in RFI between the two groups of sires was high.

Our results show clear effects due to the ewes’ parity in their response to and recovery from CHA. As expected, lambs suckling MULT ewes were heavier and grew faster (higher ADG) compared to those from PRIM ewes (Table 4; Figure 4). The parity (and the age) is well known to be among the main factors driving the adaptive capacity of the female to cope with challenging situations in their productive cycles and careers (Nielsen et al., 2003; Meikle et al., 2004; Friggens et al., 2007; González-García et al., 2014, 2015). The individual adaptive capacity of an animal facing a CHA is likely to increase with the increase in the frequency for which it is repetitively facing the same CHA. This explains differences in responses between PRIM and MULT reproductive females submitted to undernutrition events, which is consistent with our past works (González-García et al., 2014, 2015, 2020b), which is likely related to an increase in the maturity of the mechanisms to be displayed as productive life progresses.

Similarly to ewes’ parity, the litter size at pregnancy and during the suckling period is a classical factor implicated in the ‘pull effect’ for energy demands, thus affecting energy balance with more or less intensity. As expected, in our study SING lambs were heavier and grew faster (higher ADG) compared to TWIN lambs (Table 4; Figure 4). Surprisingly, we did not observe any significant effect of lamb sex on the individual BW or growth rate monitored during the experiment, but again this is likely related to the short-term nature of the CHA applied in this study.

Results concerning litter size are in agreement with our previous reports (González-García et al., 2014, 2015; González-García and Hazard, 2016). Focusing on sex effects, however, our findings are different from those observed in a larger timespan (González-García and Hazard, 2016).

### Evidence of between-animal variability in the response/recovery to the short-term challenge

There are evident between-animal differences in the responses and recoveries of ewes, which may be seen by visual inspection in most of the monitored parameters (Supplementary Figures S1 to S11). Such potential between-animal variability may provide new insights into how such short and abrupt CHA affects some key physiological parameters, and to what extent the short-term ewes’ response-recovery profiles are influenced by the individual nature of the animals. The mechanisms provoking such intra-flock variability in the physiological and behavioral responses to the CHA are complex and tightly related to the individual adaptive capacities of these ewes, which are deeply discussed elsewhere in a separate-related article (Gindri et al., 2023). Individual variability for BR dynamics, assessed through changes in BW and BCS over time, has already been investigated in this breed (Macé et al., 2019a), with several clusters of ewes identified related to different profiles of BR mobilization and accretion dynamics throughout productive cycles. Part of this individual variability in BR dynamics originated from the genetic component with high genetic relationships between mobilization and accretion of BR (Macé et al., 2019b). It may be hypothesized that individual variability in metabolic response to short-term undernutrition could have also a genetic determinism.

## Conclusions

The DMI, DFT, the monitored plasma metabolites (NEFA, β-OHB, glucose, and urea), and T3 responded well to the 2-d nutritional challenge induced to meat ewes, with observed meaningful drops (or rises) and recoveries affecting postchallenge levels of these parameters. The ewes’ parity was the main factor that influenced the rate of response to and the rate of recovery from challenge, as illustrated by DMI, DFT, β-OHB, and glucose. Lambs’ growth was affected by the parity of their challenged dams and litter size during suckling, but not sex (MULT and singleton lambs grew faster than PRIM and twins, respectively). Results suggest between-animal variability in response and recovery profiles to the challenge, for which further research is warranted. Original, relevant, and necessary quantifiable data are provided with this work in an emerging field of research.

## Supplementary Material

txad141_suppl_Supplementary_Figures_S1-S11

## Abbreviations

ADG: average daily gain
BCS: body condition score
BR: body reserves
BW: body weight
β-OHB: β-hydroxybutyrate
CHA: nutritional challenge
DFT: dorsal fat thickness
DM: dry matter
DMI: dry matter intake
DPIE: digestible proteins in the intestine when nitrogen (N) is limiting, calculated as PDIA (dietary protein undegraded in the rumen which is digestible in the small intestine) + PDIMN (microbial protein that can be synthesized from the rumen degraded dietary N when energy is not limiting)
DPIN: digestible proteins in the intestine when energy is limiting, calculated as PDIA + PDIME (microbial protein that can be synthesized from the energy available in the rumen when degraded N is not limiting)
FFV: forage fill unit value for sheep
FUL: net energy forage unit for lactation
MULT: multiparous
NEFA: non-esterified fatty acids
PRIM: primiparous
RFI: residual feed intake
T3: triiodothyronine
