# Supplementary material for: Short-term responses of meat ewes facing an acute nutritional challenge in early-mid lactation
Source: Transl Anim Sci. 2023 Dec 11;8:txad141. doi: 10.1093/tas/txad141 (PMC10782914; doi:10.1093/tas/txad141)

**Short-term responses of meat ewes facing an acute nutritional challenge at early suckling**

Eliel González-García*^1^, Marcelo Gindri§, Christian Durand†, Noëllie Lafon†, Sébastien Douls†, Gaëtan Bonafe†, Valentin Coulon†, Dominique Hazard‡, Laurent Bonnal¶, Anne Tesnière*, Irene Llach*, Sara Parisot†, and Laurence Puillet§

*SELMET, INRAE, CIRAD, L’Institut Agro Montpellier SupAgro, Univ Montpellier, 34060 Montpellier, France

†INRAE UE321 La Fage, 12250 Saint-Jean-et-Saint-Paul, France

‡INRAE UMR1388 GENPHYSE Université de Toulouse, ENVT, 31326 Castanet-Tolosan, France

§Université Paris-Saclay, INRAE, AgroParisTech, UMR Modélisation Systémique Appliquée aux Ruminants, 91120, Palaiseau, France

¶SELMET, CIRAD, INRAE, L’Institut Agro Montpellier SupAgro, Univ Montpellier, 34398 Montpellier, France

^1^Corresponding author: [eliel.gonzalez-garcia@inrae.fr](mailto:eliel.gonzalez-garcia@inrae.fr)

**Table 1**. Individual characteristics (parity, feed efficiency genetic line, litter size at lambing, BW and BCS) of the 40 experimental ewes chosen at early-mid pregnancy. The BW and BCS data were taken at mid-pregnancy (January 14^th^).

| **Number** | **EID** | **Parity** | **Age, yr** | **Genetic Line** | **Litter Size** | **BW, kg** | **BCS, 1-5** | **Lambing date** |
| --- | --- | --- | --- | --- | --- | --- | --- | --- |
| 1 | 203297 | PRIM | 2 | RFI+ | 2 | 45.5 | 2.4 | 05/04 |
| 2 | 203346 | PRIM | 2 | RFI+ | 2 | 46.0 | 2.7 | 05/04 |
| 3 | 203348 | PRIM | 2 | RFI- | 2 | 44.7 | 2.4 | 06/04 |
| 4 | 203293 | PRIM | 2 | RFI- | 2 | 43.0 | 2.7 | 07/04 |
| 5 | 183025 | MULT | 4 | RFI+ | 2 | 56.3 | 2.2 | 13/04 |
| 6 | 203173 | PRIM | 2 | RFI+ | 2 | 47.4 | 2.5 | 15/04 |
| 7 | 203333 | PRIM | 2 | RFI+ | 1 | 52.8 | 2.6 | 19/04 |
| 8 | 203004 | PRIM | 2 | RFI- | 1 | 41.4 | 2.4 | 20/04 |
| 9 | 203069 | PRIM | 2 | RFI- | 1 | 44.5 | 2.4 | 21/04 |
| 10 | 173169 | MULT | 5 | RFI- | 2 | 61.4 | 2.5 | 22/04 |
| 11 | 203511 | PRIM | 2 | RFI+ | 2 | 47.1 | 2.5 | 04/04 |
| 12 | 173488 | MULT | 5 | RFI+ | 2 | 66.2 | 2.8 | 04/04 |
| 13 | 183539 | MULT | 4 | RFI- | 2 | 55.9 | 2.3 | 06/04 |
| 14 | 183562 | MULT | 4 | RFI- | 2 | 60.2 | 2.6 | 06/04 |
| 15 | 173316 | MULT | 5 | RFI+ | 2 | 62.3 | 3.0 | 09/04 |
| 16 | 173172 | MULT | 5 | RFI- | 1 | 62.4 | 2.8 | 06/04 |
| 17 | 183021 | MULT | 4 | RFI+ | 1 | 55.9 | 2.7 | 12/04 |
| 18 | 203248 | PRIM | 2 | RFI- | 1 | 49.0 | 2.5 | 04/04 |
| 19 | 173297 | MULT | 5 | RFI+ | 2 | 63.4 | 2.4 | 13/04 |
| 20 | 173477 | MULT | 5 | RFI+ | 2 | 62.3 | 2.7 | 14/04 |
| 21 | 203056 | PRIM | 2 | RFI+ | 2 | 47.9 | 3.0 | 04/04 |
| 22 | 203230 | PRIM | 2 | RFI+ | 1 | 47.4 | 2.4 | 04/04 |
| 23 | 183454 | MULT | 4 | RFI- | 2 | 58.0 | 3.1 | 23/04 |
| 24 | 173360 | MULT | 5 | RFI- | 2 | 62.0 | 2.7 | 08/04 |
| 25 | 173264 | MULT | 5 | RFI+ | 2 | 66.0 | 2.9 | 14/04 |
| 26 | 203255 | PRIM | 2 | RFI+ | 2 | 51.2 | 3.0 | 17/04 |
| 27 | 203240 | PRIM | 2 | RFI- | 2 | 49.2 | 2.4 | 21/04 |
| 28 | 203055 | PRIM | 2 | RFI- | 1 | 46.5 | 2.5 | 20/04 |
| 29 | 203507 | PRIM | 2 | RFI+ | 1 | 43.5 | 2.3 | 22/04 |
| 30 | 183148 | MULT | 4 | RFI- | 2 | 54.0 | 2.6 | 24/04 |
| 31 | 183089 | MULT | 4 | RFI+ | 2 | 57.2 | 2.4 | 05/04 |
| 32 | 173144 | MULT | 5 | RFI- | 2 | 67.6 | 2.8 | 06/04 |
| 33 | 173555 | MULT | 5 | RFI+ | 2 | 61.3 | 2.6 | 06/04 |
| 34 | 183535 | MULT | 4 | RFI- | 2 | 62.3 | 3.2 | 06/04 |
| 35 | 203379 | PRIM | 2 | RFI- | 2 | 40.2 | 2.4 | 09/04 |
| 36 | 203352 | PRIM | 2 | RFI+ | 1 | 48.5 | 2.7 | 04/04 |
| 37 | 203250 | PRIM | 2 | RFI- | 1 | 48.9 | 2.4 | 02/04 |
| 38 | 183186 | MULT | 4 | RFI+ | 2 | 72.0 | 2.5 | 12/04 |
| 39 | 183578 | MULT | 4 | RFI- | 1 | 63.4 | 3.3 | 06/04 |
| 40 | 203326 | PRIM | 2 | RFI- | 2 | 55.1 | 3.0 | 14/04 |

Average lambing date for these ewes was April 10 (±7 d). EID: individual electronic identification; RFI+: inefficient ewe; RFI-: efficient ewe

**Figure S1.** Time trends of the individual response-recovery profiles for **DMI (kg/d)** of Romane ewes (primiparous, PRIM, or multiparous, MULT; efficient, RFI-, or inefficient, RFI+), when facing a 2-d nutritional challenge during suckling (i.e. 29 ±6.8 days relative to lambing).


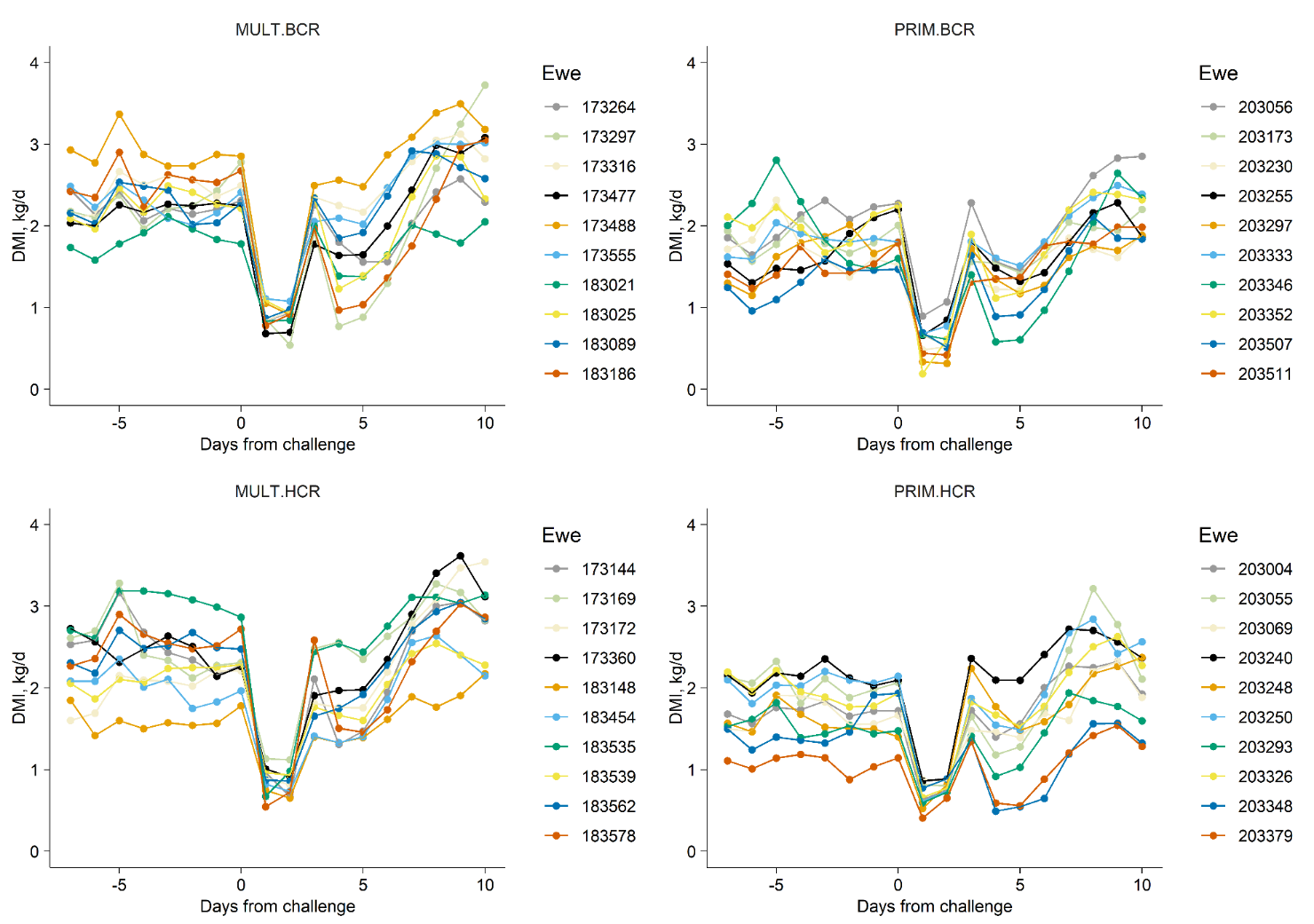


**MULT×RFI+**

**PRIM×RFI+**

**PRIM×RFI-**

**MULT×RFI-**

**Figure S2.** Time trends of the individual response-recovery profiles for **DMI (g/kg BW)** of Romane ewes (primiparous, PRIM, or multiparous, MULT; efficient, RFI-, or inefficient, RFI+), when facing a 2-d nutritional challenge during suckling (i.e. 29 ±6.8 days relative to lambing).


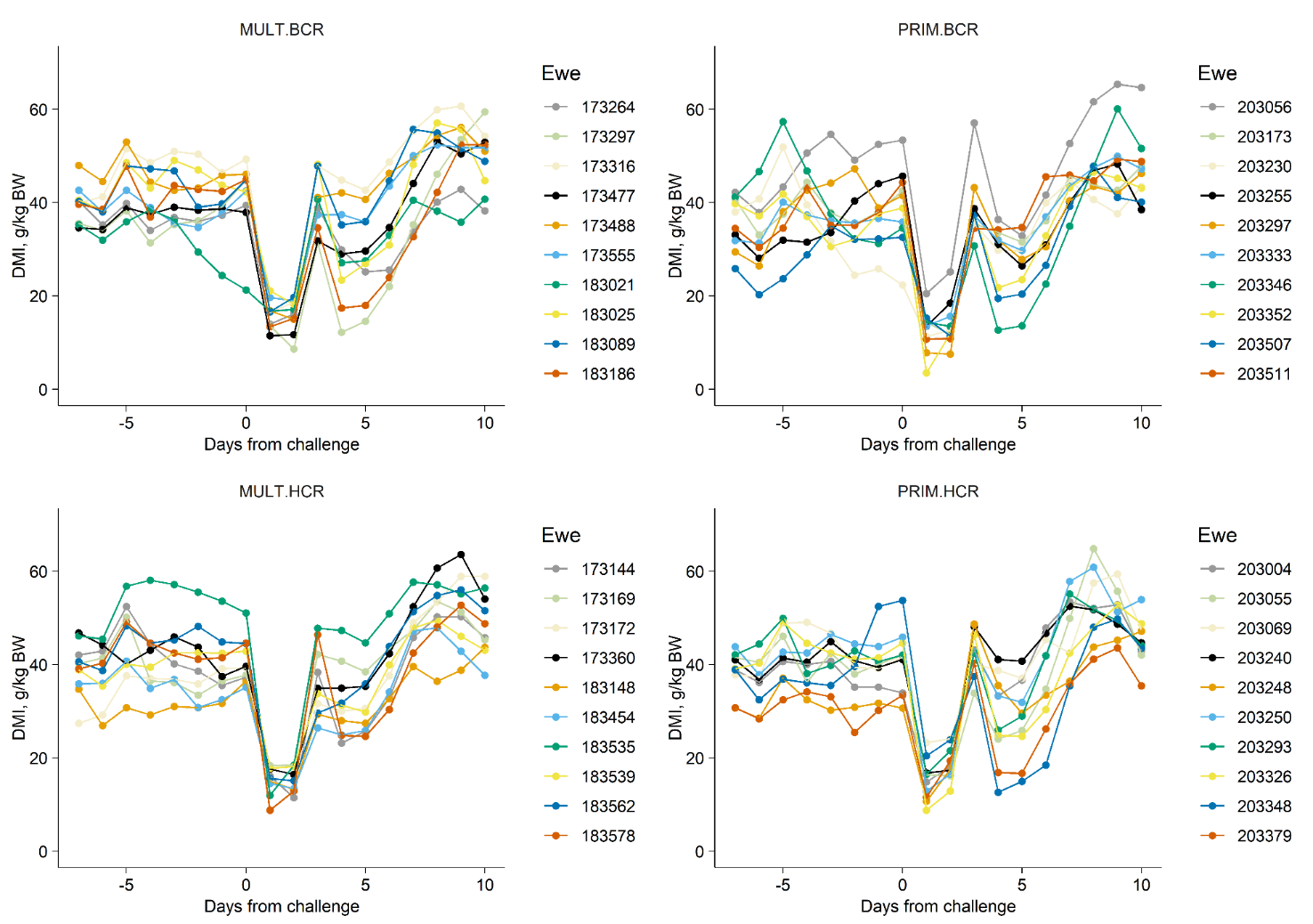


**MULT×RFI+**

**PRIM×RFI+**

**PRIM×RFI-**

**MULT×RFI-**

**Figure S3.** Time trends of the individual response-recovery profiles for **DMI (g/kg BW^.75^)** of Romane ewes (primiparous, PRIM, or multiparous, MULT; efficient, RFI-, or inefficient, RFI+), when facing a 2-d nutritional challenge during suckling (i.e. 29 ±6.8 days relative to lambing).


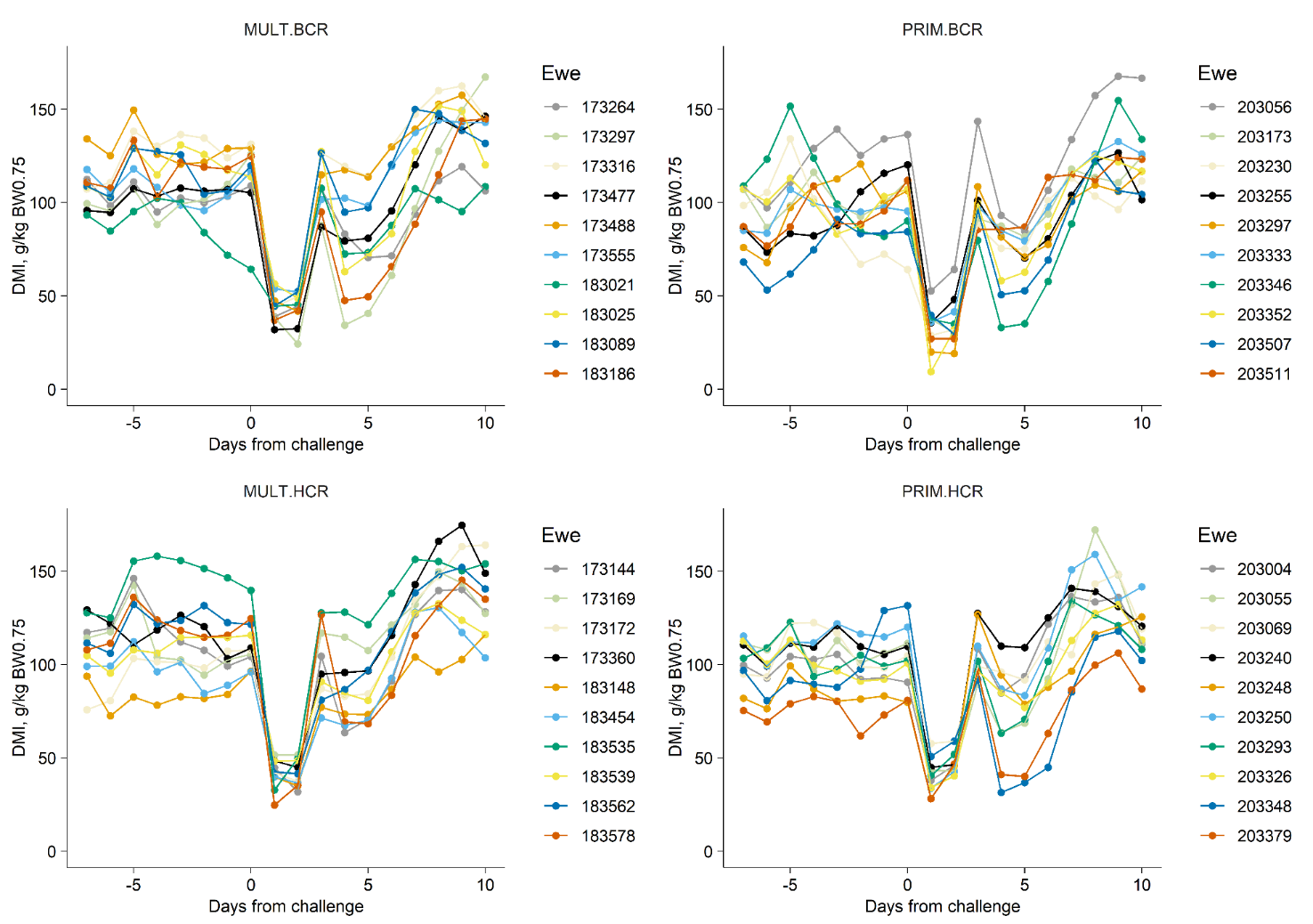


**MULT×RFI+**

**PRIM×RFI+**

**PRIM×RFI-**

**MULT×RFI-**

**Figure S4.** Time trends of the individual response-recovery profiles for **BW (kg)** of Romane ewes (primiparous, PRIM, or multiparous, MULT; efficient, RFI-, or inefficient, RFI+), when facing a 2-d nutritional challenge during suckling (i.e. 29 ±6.8 days relative to lambing).


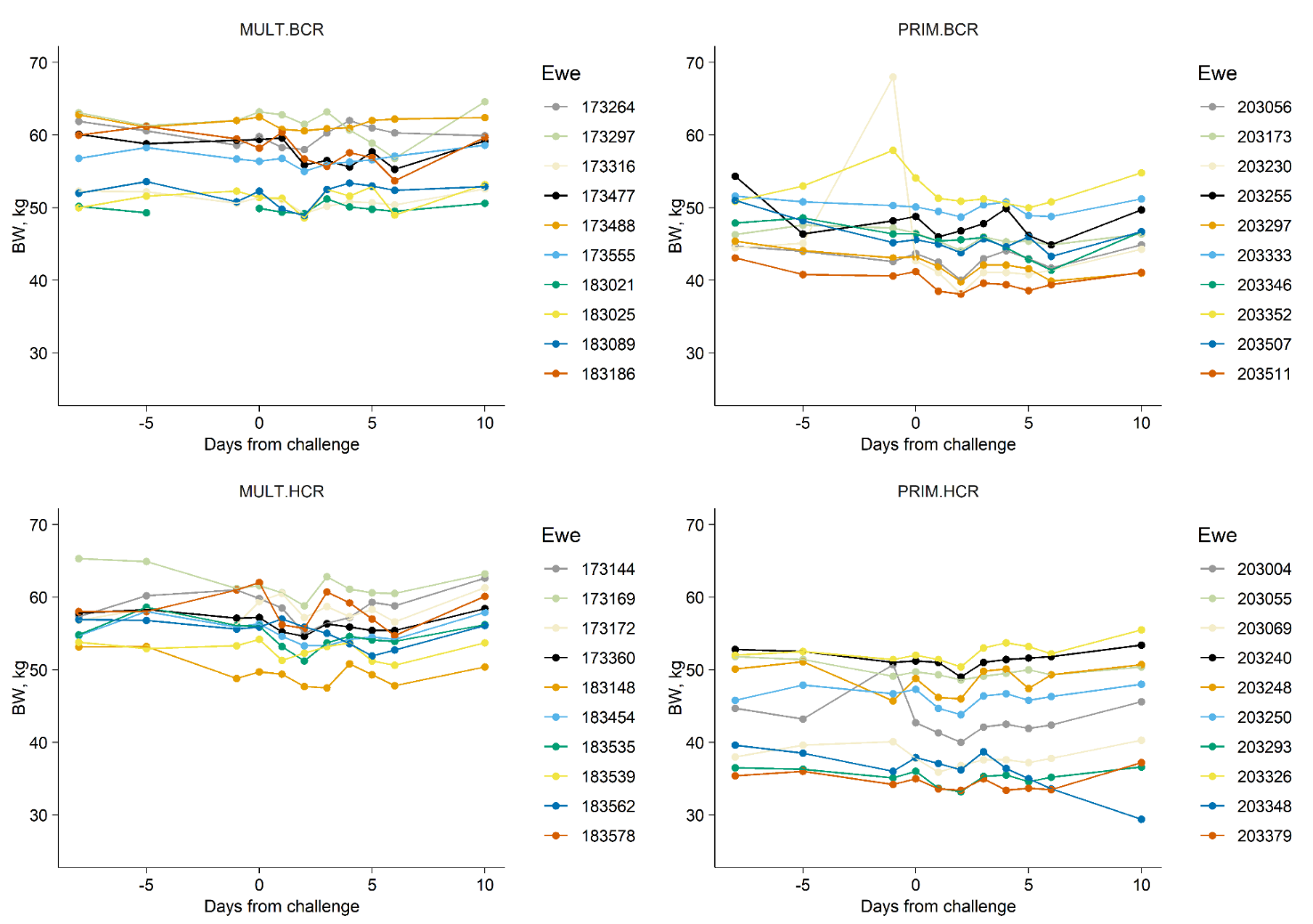


**MULT×RFI+**

**PRIM×RFI+**

**PRIM×RFI-**

**MULT×RFI-**

**Figure S5.** Time trends of the individual response-recovery profiles for **BCS (1-5)** of Romane ewes (primiparous, PRIM, or multiparous, MULT; efficient, RFI-, or inefficient, RFI+), when facing a 2-d nutritional challenge during suckling (i.e. 29 ±6.8 days relative to lambing).


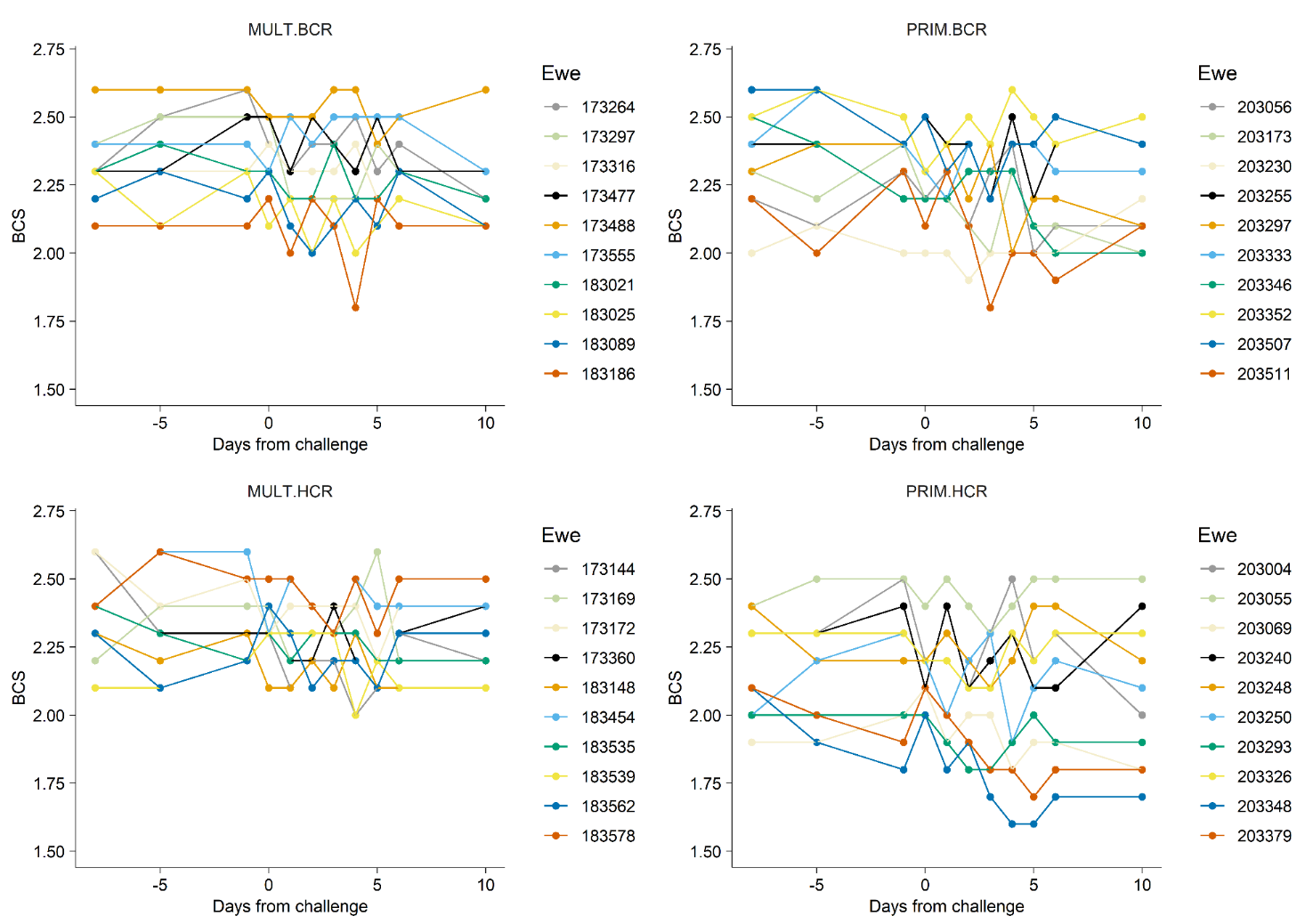


**MULT×RFI+**

**PRIM×RFI+**

**PRIM×RFI-**

**MULT×RFI-**

**Figure S6.** Time trends of the individual response-recovery profiles for **DFT (cm)** of Romane ewes (primiparous, PRIM, or multiparous, MULT; efficient, RFI-, or inefficient, RFI+), when facing a 2-d nutritional challenge during suckling (i.e. 29 ±6.8 days relative to lambing).


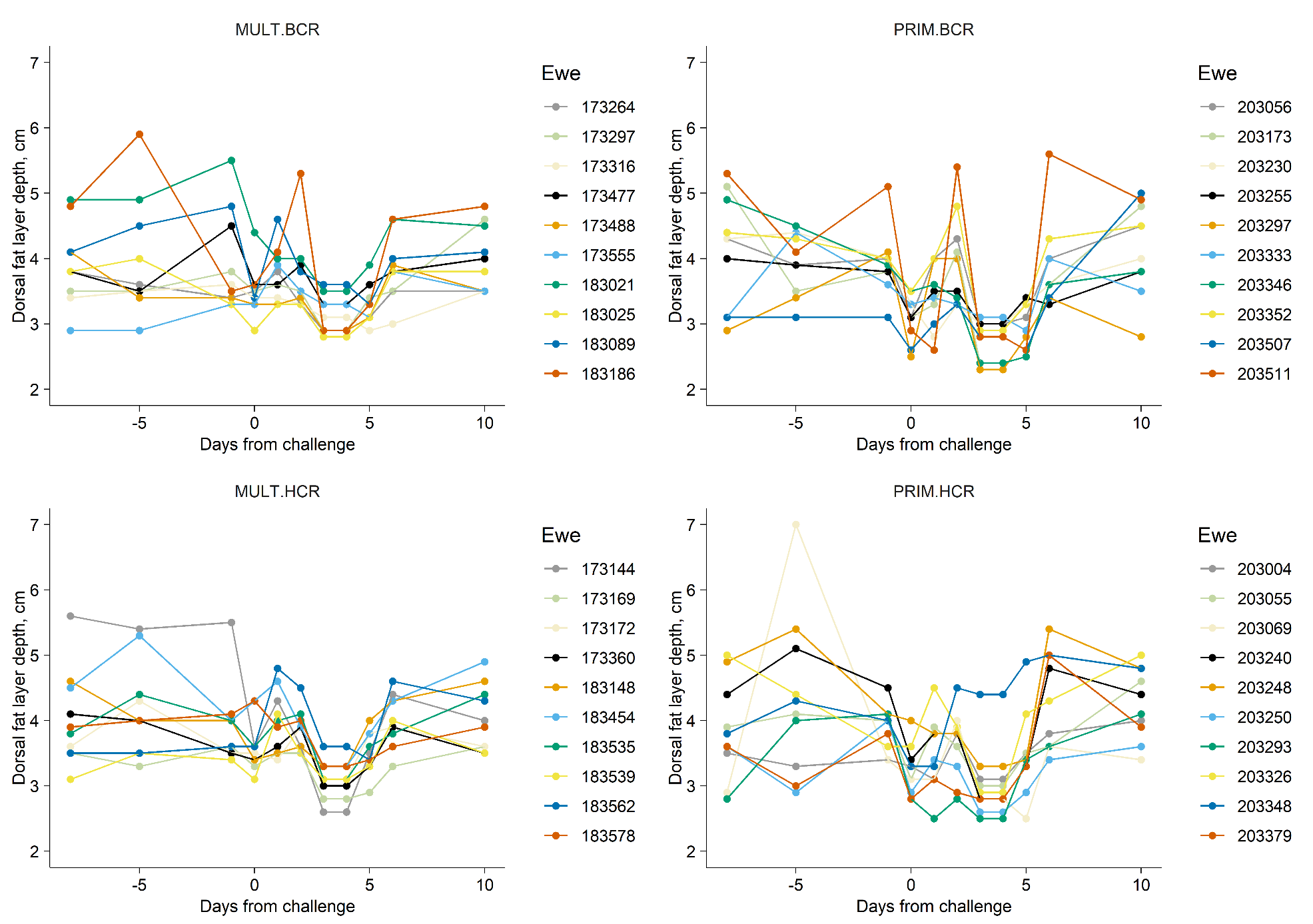


**MULT×RFI+**

**PRIM×RFI+**

**PRIM×RFI-**

**MULT×RFI-**

**Figure S7.** Time trends of the individual response-recovery profiles for **β-OHB (mg/L)** of Romane ewes (primiparous, PRIM, or multiparous, MULT; efficient, RFI-, or inefficient, RFI+), when facing a 2-d nutritional challenge during suckling (i.e. 29 ±6.8 days relative to lambing).


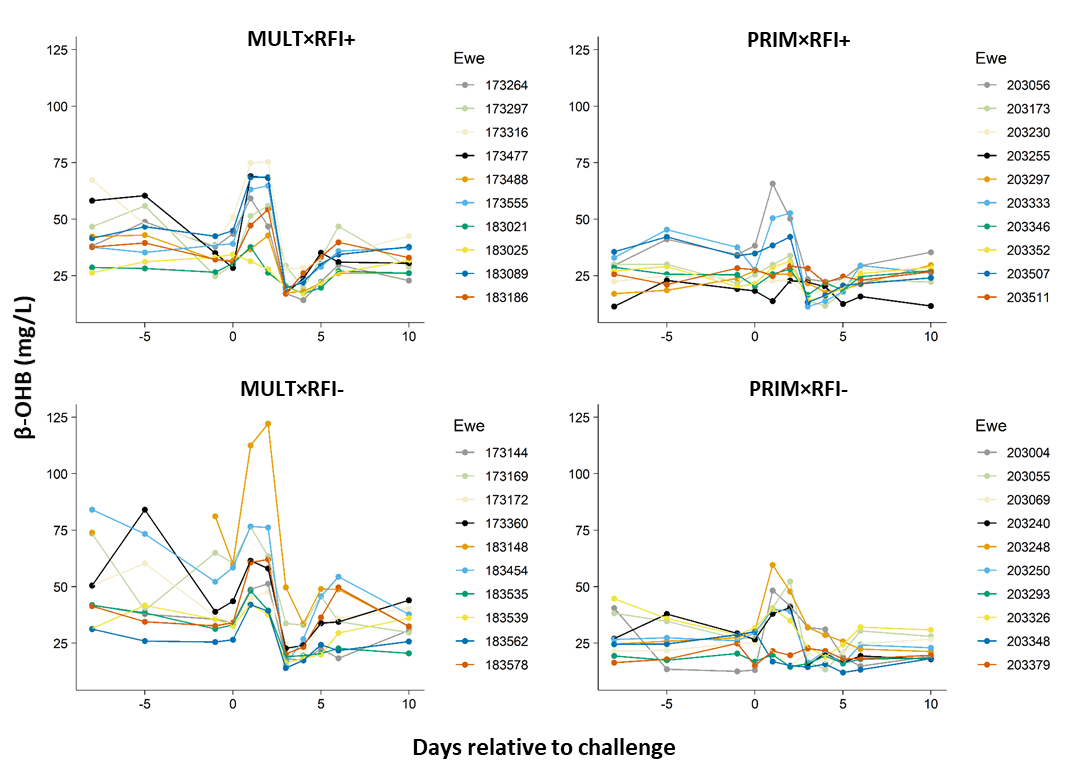


**Figure S8.** Time trends of the individual response-recovery profiles for plasma **glucose (g/L)** of Romane ewes (primiparous, PRIM, or multiparous, MULT; efficient, RFI-, or inefficient, RFI+), when facing a 2-d nutritional challenge during suckling (i.e. 29 ±6.8 days relative to lambing).


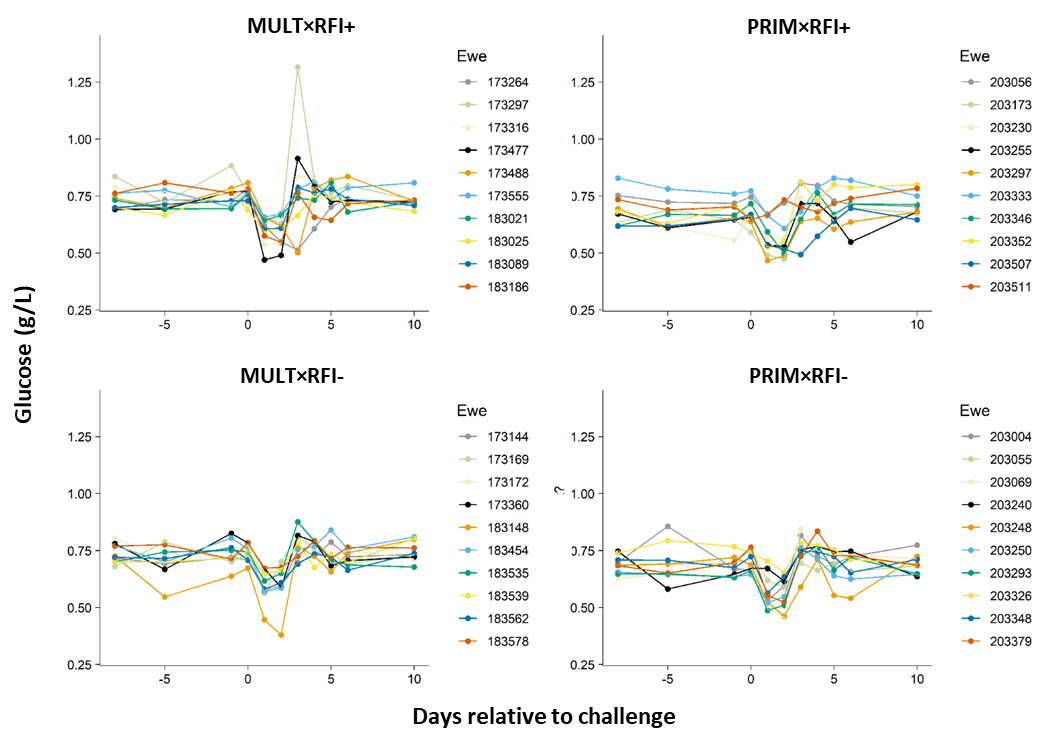


**Figure S9.** Time trends of the individual response-recovery profiles for plasma **T3 (ng/dL)** of Romane ewes (primiparous, PRIM, or multiparous, MULT; efficient, RFI-, or inefficient, RFI+), when facing a 2-d nutritional challenge during suckling (i.e. 29 ±6.8 days relative to lambing).


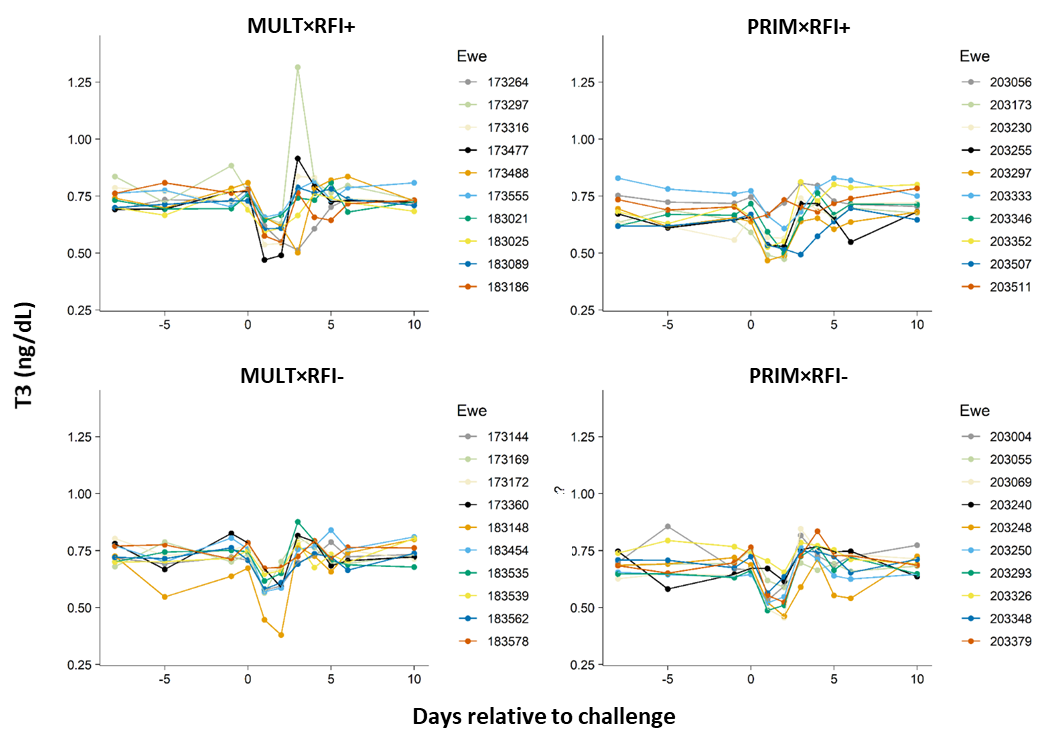


**Figure S10.** Time trends of the individual response-recovery profiles for plasma **urea (g/L)** of Romane ewes (primiparous, PRIM, or multiparous, MULT; efficient, RFI-, or inefficient, RFI+), when facing a 2-d nutritional challenge during suckling (i.e. 29 ±6.8 days relative to lambing).


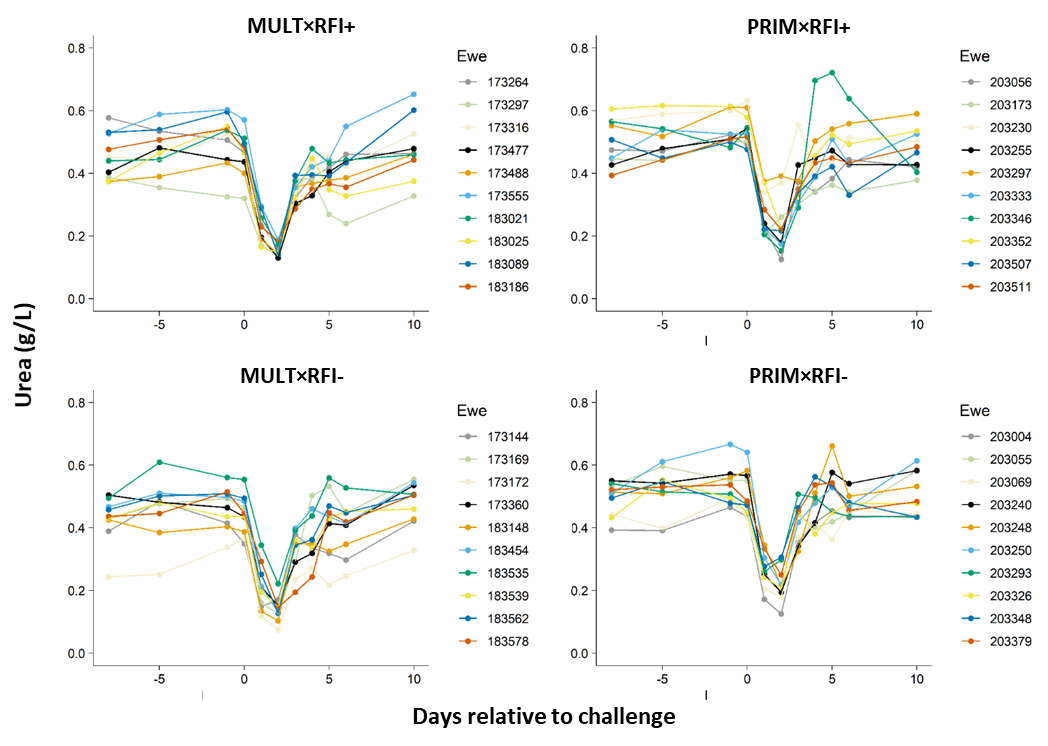


**Figure S11.** Time trends of the lambs BW (kg) progression throughout the experiment, around a 2-d nutritional challenge during suckling (i.e. 29 ±6.8 days relative to lambing) faced by their dams (Romane ewes; primiparous, PRIM, or multiparous, MULT; efficient, RFI-, or inefficient, RFI+).


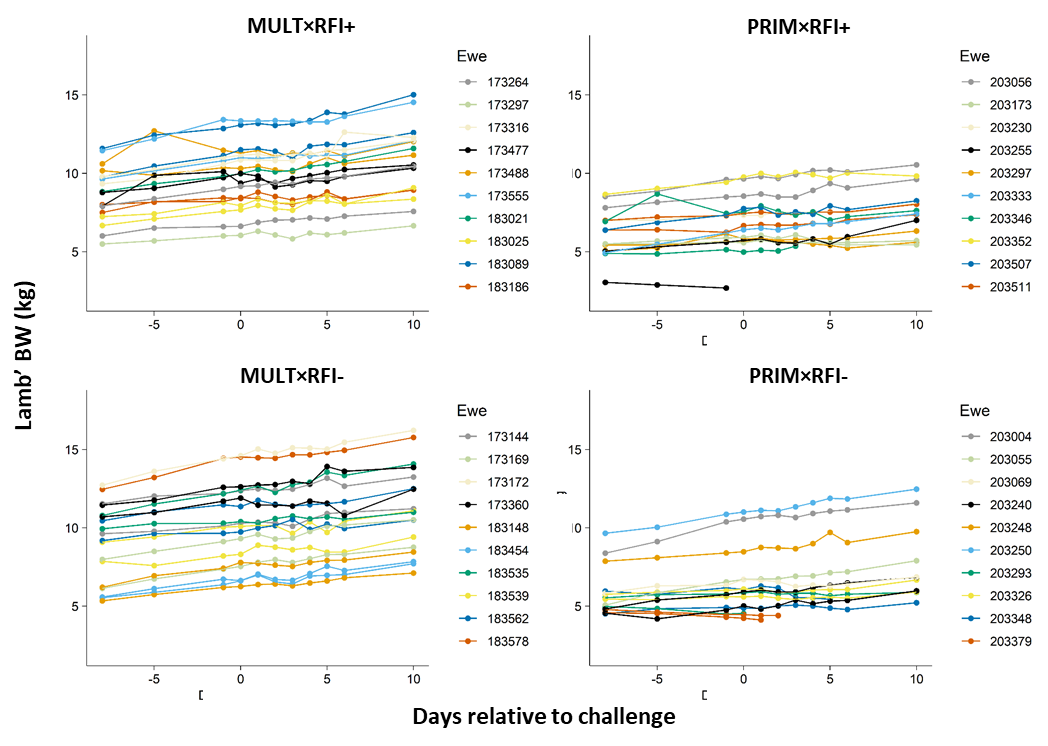

Supplement: txad141_suppl_Supplementary_Figures_S1-S11 [file txad141_suppl_supplementary_figures_s1-s11.docx]
